# Plasma HPV ctDNA as a dynamic biomarker for treatment response and prognosis in locally advanced cervical cancer: protocol for a prospective cohort study

**DOI:** 10.3389/fonc.2026.1844938

**Published:** 2026-07-22

**Authors:** Hailing Hou, Li Zhang, Chen Li, Miao Liu

**Affiliations:** 1 Department of Radiation Oncology, Tianjin’s Clinical Research Center for Cancer, Key Laboratory of Cancer Prevention and Therapy, Tianjin Medical University Cancer Institute & Hospital, National Clinical Research Center for Cancer, Tianjin, China; 2 Gynecologic Oncology Department, Tianjin’s Clinical Research Center for Cancer, Key Laboratory of Cancer Prevention and Therapy, Tianjin Medical University Cancer Institute & Hospital, National Clinical Research Center for Cancer, Tianjin, China

**Keywords:** biomarker, cervical cancer, chemoradiotherapy, circulating tumor DNA, human papillomavirus, liquid biopsy

## Abstract

**Background:**

Locally advanced cervical cancer (LACC) remains a major global health burden, and current imaging-based assessment after standard concurrent chemoradiotherapy (CCRT) is limited by delayed detection of treatment failure and poor sensitivity for microscopic residual disease. Circulating human papillomavirus DNA (HPV ctDNA) represents a tumor-specific biomarker with potential for real-time treatment monitoring. This prospective cohort study aims to evaluate whether dynamic monitoring of plasma HPV ctDNA can predict treatment response and prognosis in patients with LACC receiving CCRT.

**Methods/design:**

This single-center, observational cohort study will enroll 57 patients with histologically confirmed LACC (FIGO 2018 stage IB2–IVA) and baseline high-risk HPV infection. All patients will receive standard CCRT including external beam radiotherapy (45–50.4 Gy), concurrent weekly cisplatin (40 mg/m²), and intracavitary brachytherapy. Peripheral blood samples (10 mL) will be collected at five predefined time points: baseline (T0), mid-treatment (T1), end of CCRT (T2), 3 months post-CCRT (T3), and every 6 months thereafter for 24 months. Plasma HPV ctDNA will be quantified using droplet digital PCR targeting HPV16/18 E6/E7 genes. The primary endpoint is progression-free survival (PFS), comparing patients with detectable versus undetectable ctDNA at 3 months post-CCRT.

**Conclusion:**

This study will provide prospective evidence for HPV ctDNA as a minimally invasive biomarker for treatment monitoring and prognostic stratification in LACC, potentially informing future risk-adapted therapeutic strategies.

## Introduction

Cervical cancer remains a substantial global health challenge, with an estimated 662,044 new cases and 348,709 deaths occurring worldwide in 2022, representing the fourth most common malignancy and cause of cancer-related mortality in women (1). The burden disproportionately affects low- and middle-income countries, where 90% of cervical cancer deaths occur, primarily reflecting inequities in access to human papillomavirus (HPV) vaccination, screening programs, and definitive treatment services (2). Notably, China and India together account for 42% of global incident cases and 39% of deaths, with projections indicating a 56.8% increase in new cases and an 80.7% rise in mortality by 2050 if current rates remain unchanged (1).

Locally advanced cervical cancer (LACC), encompassing International Federation of Gynecology and Obstetrics (FIGO) 2018 stages IB2 through IVA, constitutes a considerable proportion of newly diagnosed cases in resource-limited settings (3). Concurrent chemoradiotherapy (CCRT) with platinum-based chemotherapy has been established as the standard therapeutic approach for LACC since 1999, demonstrating significant survival benefits in landmark randomized controlled trials (4). Despite advances in treatment delivery techniques including intensity-modulated radiotherapy and image-guided brachytherapy, approximately 30-40% of patients receiving CCRT experience treatment failure, manifesting as incomplete response, residual disease, or early recurrence within two years (5, 6). This substantial recurrence rate underscores the urgent need for reliable biomarkers capable of identifying high-risk patients who may benefit from treatment intensification or alternative therapeutic strategies.

Circulating tumor DNA (ctDNA) has emerged as a promising liquid biopsy approach for detecting molecular residual disease, monitoring tumor dynamics, and predicting clinical outcomes across diverse malignancies. In HPV-driven cancers, circulating HPV DNA represents a uniquely tumor-specific biomarker with exceptional analytical specificity, as integrated viral DNA sequences persist exclusively in malignant cells and are absent from normal tissues or high-grade precancerous lesions (7). Recent technological advances in droplet digital polymerase chain reaction (ddPCR) and next-generation sequencing (NGS) platforms enable highly sensitive quantification of HPV ctDNA, with analytical limits of detection approaching 5 copies per milliliter of plasma (8). Accumulating evidence from head and neck squamous cell carcinoma, anal carcinoma, and emerging cervical cancer cohorts demonstrates strong correlations between HPV ctDNA levels and tumor burden, treatment response kinetics, and disease recurrence risk (9, 10). Critically, persistent detectability of HPV ctDNA after chemoradiotherapy has been associated with dramatically inferior progression-free survival compared to molecular clearance, with hazard ratios exceeding 4.0 in multiple independent cohorts (11). A multicenter validation study reported 2-year PFS of 82% versus 15% for patients with undetectable versus detectable HPV ctDNA at 4–6 weeks post-CCRT (9). A meta-analysis encompassing 706 cervical cancer patients demonstrated that detectable ctDNA during treatment conferred substantially elevated risks for progression and mortality (12).

Despite these promising findings, prospective evidence evaluating HPV ctDNA as a dynamic biomarker specifically in LACC patients undergoing CCRT remains limited. Most existing studies are retrospective, utilize heterogeneous sampling schedules, employ non-standardized detection methodologies, or combine patients across diverse treatment paradigms, thereby constraining the generalizability and clinical applicability of findings (13–15). Besides, critical knowledge gaps persist regarding optimal timing of ctDNA assessments, prognostic thresholds for risk stratification, concordance with imaging-based response evaluation, and the independent predictive value of ctDNA status when adjusted for established clinicopathological risk factors. The present prospective cohort study aims to address these evidence gaps by evaluating whether dynamic monitoring of plasma HPV ctDNA can predict treatment response and prognosis in patients with LACC receiving standard CCRT.

## Methods and analysis

### Study design and patients

**Figure 1:**
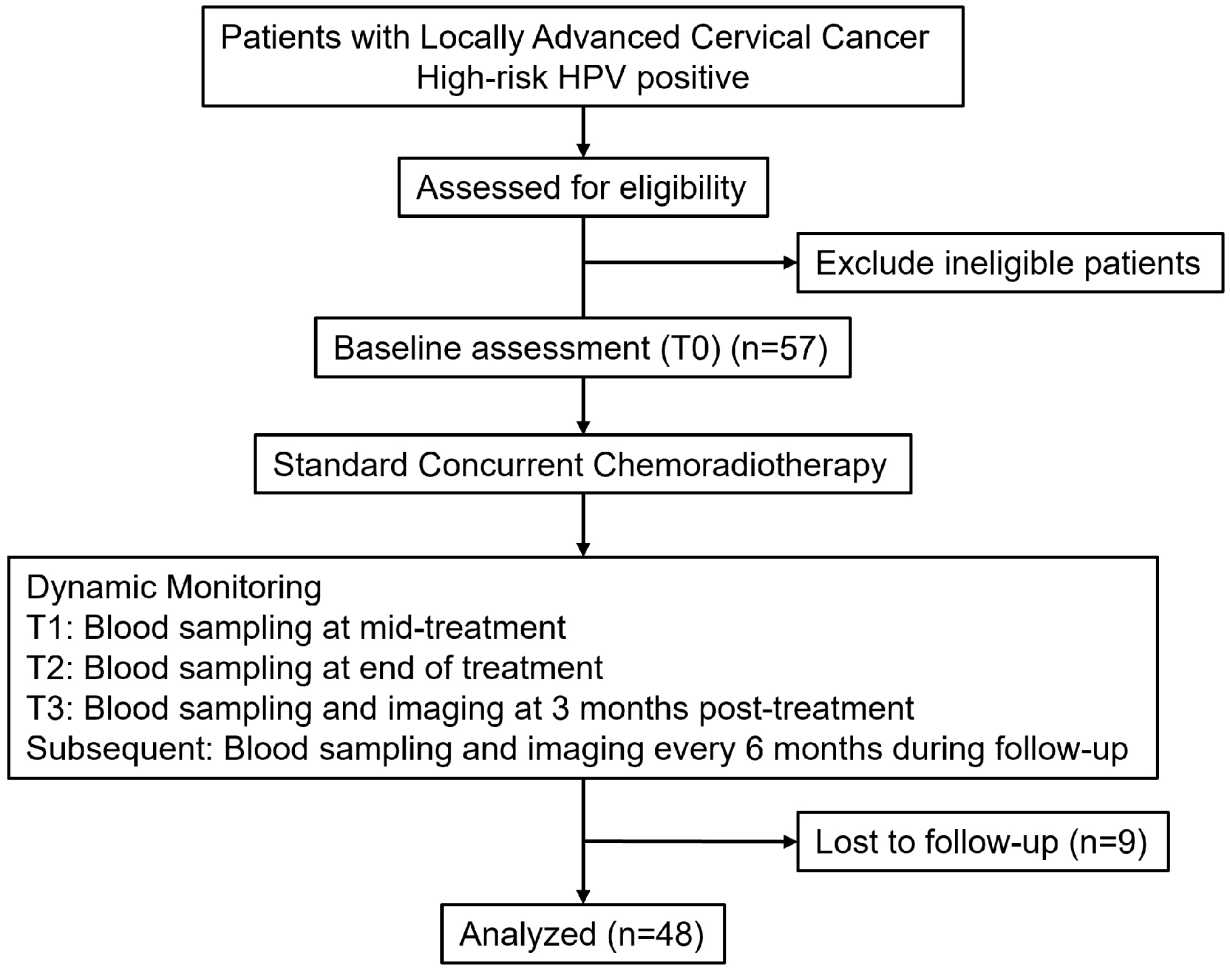
Study flow diagram.

This prospective, single-center, observational cohort study will be conducted at the Department of Radiation Oncology, Tianjin Medical University Cancer Institute and Hospital, Tianjin, China, with patient recruitment planned to begin in January 2027 and continue for a 2-year period through December 2028. The hospital is a leading tertiary cancer center with advanced radiotherapy imaging facilities, enabling a multimodal image-guided radiotherapy system to improve target delineation and treatment planning, supporting high-quality clinical research. The study protocol was developed in accordance with the Declaration of Helsinki and adheres to the Standard Protocol Items: Recommendations for Interventional Trials (SPIRIT) guidelines for observational cohort studies. Ethical approval has been obtained from the Institutional Review Board of Tianjin Medical University Cancer Institute and Hospital (approval number: bc20254101). The study has been registered at ClinicalTrials.gov under the identifier of ChiCTR2600120726. All participants will provide written informed consent prior to enrollment. The overview of the study design and participant flow is illustrated in **Figure 1**.

Eligible participants will be consecutively recruited from patients diagnosed with HPV-associated cervical cancer at our institution. Study-related examinations and blood sampling will be performed according to the protocol schedule outlined in **Table 1**. Importantly, this study does not involve investigational drugs or experimental interventions. All patients will receive standard-of-care CCRT according to National Comprehensive Cancer Network (NCCN) guidelines and Chinese Society of Clinical Oncology (CSCO) recommendations. No study medications will be provided free of charge, and no financial compensation or additional economic incentives will be offered to participants, treating physicians, or research staff for trial participation.

**Table 1:** Schedule of enrolment, interventions, and assessments.

Study period	Enrolment	Allocation	Treatment & monitoring			Post-treatment follow-up		
Procedure/assessment	Screening (-t_1_)	Baseline (T0)	Week 3-4 (T1)	End CCRT (T2)	3 months (T3)	6 months	12 months	18–24 months
Enrolment								
Eligibility screening	X							
Informed consent	X							
Allocation		X						
Medical history	X							
Assessments								
Demographics/baseline	X	X						
Physical examination	X	X	X	X	X	X	X	X
ECOG performance status	X	X	X	X	X	X	X	X
Vital signs (BP HR T)	X	X	X	X	X	X	X	X
Concomitant medications	X	X	X	X	X	X	X	X
Laboratory tests	X	X		X	X	X	X	X
HPV DNA testing (cervical)	X							
Imaging								
Pelvic MRI	X	X			X	X	X	X
PET-CT (if indicated)	X				X	X	X	X
Treatment								
CCRT administration		X	X	X				
Treatment adherence		X	X	X				
Blood sampling								
Plasma HPV ctDNA		X	X	X	X	X	X	X
ctDNA analysis								
ddPCR (all samples)		X	X	X	X	X	X	X
NGS (subset)		X		X	X			
Response assessment								
RECIST 1.1 evaluation					X	X	X	X
Safety & qol								
Adverse event monitoring		X	X	X	X	X	X	X
EORTC QLQ-C30/CX24		X		X	X	X	X	X
Data management								
CRF completion	X	X	X	X	X	X	X	X
Data verification					X		X	X
Query resolution	Ongoing throughout study period							

BP, blood pressure; CCRT, concurrent chemoradiotherapy; CRF, case report form; ct, circulating tumor; ddPCR, droplet digital PCR; DNA, deoxyribonucleic acid; ECOG, Eastern Cooperative Oncology Group; EORTC, European Organization for Research and Treatment of Cancer; HPV, human papillomavirus; HR, heart rate; MRI, magnetic resonance imaging; NGS, next-generation sequencing; PET-CT, positron emission tomography-computed tomography; QLQ, Quality of Life Questionnaire; QOL, quality of life; RECIST, Response Evaluation Criteria in Solid Tumors; T, temperature.

The study team consists of gynecologic oncologists responsible for initial diagnosis and staging, radiation oncologists who will deliver CCRT and assess treatment response, medical oncologists who will manage chemotherapy administration, molecular laboratory specialists who will perform ctDNA analyses, biostatisticians who will conduct data analysis, and trained research nurses responsible for patient recruitment, blood sample collection and processing, data collection, and quality control monitoring.

### Study sample and enrollment

#### Inclusion criteria

Participants will be eligible for inclusion if they meet all of the following criteria:

1. female patients aged 18–75 years;
2. histologically confirmed cervical squamous cell carcinoma, adenocarcinoma, or adenosquamous carcinoma based on biopsy specimens reviewed by experienced pathologists;
3. LACC staged as International Federation of Gynecology and Obstetrics (FIGO) 2018 classification stage IB2, IB3, II, III, or IVA, confirmed by clinical examination and imaging studies including pelvic magnetic resonance imaging (MRI) and/or positron emission tomography-computed tomography (PET-CT) (16);
4. baseline confirmation of high-risk HPV infection (types 16 and 18) by quantitative HPV DNA testing performed on cervical tissue or liquid-based cytology samples using polymerase chain reaction (PCR)-based assays (17);
5. planned to receive and medically able to complete definitive CCRT with curative intent;
6. Eastern Cooperative Oncology Group (ECOG) performance status 0–1;
7. adequate bone marrow function (white blood cell count ≥3.0×10^9^/L, absolute neutrophil count ≥1.5×10^9^/L, platelet count ≥100×10^9^/L, hemoglobin ≥90 g/L), adequate hepatic function (total bilirubin ≤1.5 times upper limit of normal [ULN], alanine aminotransferase and aspartate aminotransferase ≤2.5 times ULN), and adequate renal function (serum creatinine ≤1.5 times ULN or calculated creatinine clearance ≥60 mL/min);
8. estimated life expectancy ≥6 months;
9. ability to comply with the study protocol and scheduled follow-up visits and provision of written informed consent.

#### Exclusion criteria

Participants will be excluded if they meet any of the following conditions:

1. prior receipt of surgery, chemotherapy, radiotherapy, immunotherapy, or targeted therapy for cervical cancer;
2. non-epithelial cervical malignancies or rare histological subtypes such as neuroendocrine carcinoma, small cell carcinoma, clear cell carcinoma, or sarcoma;
3. known distant metastases at the time of enrollment confirmed by imaging studies;
4. contraindications to radiotherapy (such as prior pelvic radiation, active inflammatory bowel disease, or connective tissue disorders affecting the pelvis) or chemotherapy (such as severe hypersensitivity to platinum compounds);
5. history of other malignancies within the past 5 years, except for adequately treated basal cell or squamous cell carcinoma of the skin, carcinoma *in situ* of the cervix, or carcinoma *in situ* of the breast;
6. pregnancy confirmed by serum or urine β-human chorionic gonadotropin test, or lactation;
7. severe psychiatric disorders or cognitive impairments that would impair the ability to understand the study requirements or provide informed consent;
8. active, uncontrolled infections requiring systemic antibiotic, antifungal, or antiviral therapy;
9. significant cardiovascular disease including New York Heart Association class III or IV heart failure, unstable angina, myocardial infarction within 6 months, or serious cardiac arrhythmias;
10. participation in other interventional clinical trials during the study period that could potentially interfere with study assessments or outcomes.

#### Withdrawal criteria

Participants may be withdrawn from the study under the following circumstances:

1. voluntary withdrawal of informed consent for any reason at any time without prejudice to future medical care;
2. inability to complete the planned treatment regimen due to unacceptable toxicity or deteriorating performance status;
3. inability to comply with the follow-up schedule or protocol requirements as judged by the investigator;
4. occurrence of severe adverse events (SAEs) requiring permanent discontinuation of CCRT, such as grade 4 hematologic toxicity, grade 3–4 non-hematologic toxicity not manageable with supportive care, or life-threatening complications;
5. major protocol violations that compromise data integrity or patient safety, such as failure to obtain required blood samples at critical time points or deviation from prescribed treatment regimens;
6. development of disease progression requiring change in treatment approach during the planned CCRT course;
7. investigator’s judgment that continued participation is not in the patient’s best interest based on medical, safety, or ethical considerations.

Data collected from withdrawn participants up to the point of withdrawal will be included in the analysis according to the intention-to-treat principle unless consent for data use is specifically withdrawn.

### Treatment protocol

All enrolled patients will receive definitive CCRT as the initial treatment according to institutional standard protocols based on international guidelines. External beam radiotherapy (EBRT) will be delivered using 6-megavolt (MV) X-ray volumetric modulated arc therapy (VMAT) technique on a linear accelerator. Target volume delineation will be performed according to the European Society for Medical Oncology (ESMO) Clinical Practice Guidelines for cervical cancer management. The gross tumor volume (GTV) will include the primary cervical tumor and any involved lymph nodes identified on MRI and/or PET-CT. The clinical target volume (CTV) will encompass the GTV plus the parametria, upper vagina (at least 3 cm below the distal extent of the tumor or to the introitus if more distal involvement), uterus, and regional lymph node areas including the common iliac, internal iliac, external iliac, obturator, presacral, and para-aortic lymph nodes (if involved). The planning target volume (PTV) will be created by adding appropriate margins (typically 5–7 mm) to the CTV to account for setup uncertainties and organ motion.

The prescribed dose to 95% of the PTV will be 45–50.4 gray (Gy) administered in 1.8 Gy per fraction, delivered five fractions per week over approximately 5–6 weeks. For patients with FIGO 2018 stage IIIC1 (pelvic lymph node metastases) or IIIC2 (para-aortic lymph node metastases) disease, a simultaneous integrated boost (SIB) technique will be employed to deliver escalated doses to macroscopically involved lymph nodes. Specifically, 95% of the planning gross tumor volume of involved nodes (PGTV_nd) will receive 59.36 Gy in 2.12 Gy per fraction over the same treatment period, achieving a biologically equivalent dose appropriate for nodal disease control while maintaining acceptable doses to organs at risk.

Concurrent platinum-based chemotherapy will consist of cisplatin administered intravenously at a dose of 40 milligrams per square meter (mg/m²) body surface area weekly during the course of EBRT, with a maximum of six cycles. Chemotherapy will be administered on the same day as radiotherapy when possible, typically on Mondays. Dose modifications or delays will be implemented according to standard institutional protocols based on hematologic and non-hematologic toxicity criteria per Common Terminology Criteria for Adverse Events (CTCAE) version 5.0. Specifically, chemotherapy will be held if absolute neutrophil count is<1.5×10^9^/L, platelet count is<75×10^9^/L, or serum creatinine is >1.5 times ULN, and will be resumed when counts recover.

Intracavitary brachytherapy will be initiated in the fourth or fifth week of pelvic EBRT, after the primary tumor has shown adequate response to allow safe applicator insertion. Brachytherapy will be delivered using computed tomography (CT)-guided three-dimensional image-guided adaptive brachytherapy with an iridium-192 (Ir-192) high-dose-rate remote afterloading system. Applicators (tandem and ovoid or tandem and ring) will be placed under appropriate anesthesia, and CT imaging will be performed with the applicator in place for treatment planning. Target volumes will be delineated according to the Groupe Européen de Curiethérapie and European Society for Therapeutic Radiology and Oncology (GEC-ESTRO) recommendations, including the high-risk CTV (HR-CTV) encompassing gross residual disease and the intermediate-risk CTV (IR-CTV). A prescription dose of 7 Gy per fraction to the HR-CTV will be delivered once weekly for a total of four fractions over 2 weeks, with dose-volume constraints respected for organs at risk (bladder, rectum, sigmoid, bowel). The cumulative biologically equivalent dose in 2-Gy fractions (EQD2) for the combination of EBRT and brachytherapy will target ≥85 Gy for HR-CTV and<90 Gy for the bladder and rectum (D2cc).

### Blood sampling and time points for ctDNA detection

Peripheral venous blood samples (10 milliliters [mL] per time point) will be collected into ethylenediaminetetraacetic acid (EDTA)-anticoagulated tubes (Becton Dickinson, Franklin Lakes, New Jersey, USA) at the following predefined time points to capture ctDNA dynamics throughout the treatment course and follow-up period:

T0 (baseline): Within 7 days before initiation of CCRT. This baseline sample will establish the pretreatment HPV ctDNA level as a reference point and will be correlated with baseline tumor burden assessed by imaging and clinical examination.

T1 (mid-treatment): After 3–4 weeks of CCRT (approximately 15–20 fractions of radiotherapy and 3–4 cycles of concurrent chemotherapy).

T2 (end of CCRT): Within 1 week after completion of all CCRT including brachytherapy.

T3 (first evaluation): At 3 months (± 2 weeks) after completion of CCRT, coinciding with the first scheduled imaging assessment.

Follow-up sampling: Every 6 months (± 4 weeks) thereafter for up to 24 months from the completion of CCRT, or until disease progression, death, withdrawal of consent, or study completion, whichever occurs first. Additional blood samples may be collected at unscheduled visits if disease recurrence or progression is clinically suspected, to correlate ctDNA status with disease status.

Blood samples will be processed according to a standardized protocol to ensure consistency and minimize preanalytical variability. Specifically, blood samples must be maintained at room temperature and processed within 2 hours of collection to prevent cell lysis and release of genomic DNA that could dilute ctDNA signals. Samples will be centrifuged at 1,600 × g for 10 minutes at 4 °C to separate plasma from cellular components. The plasma supernatant will be carefully transferred to a new tube without disturbing the buffy coat layer and will be subjected to a second centrifugation at 16,000 × g for 10 minutes at 4 °C to remove any residual cells and debris. The resulting cell-free plasma will be aliquoted into 1-mL cryovials, labeled with unique identifiers, and stored immediately at –80 °C until batch analysis. All samples will be tracked using a laboratory information management system to ensure chain of custody and prevent sample mix-ups.

### ctDNA detection methods

#### DNA extraction

Circulating cell-free DNA (cfDNA) will be extracted from 1–4 mL of frozen plasma samples using the QIAamp Circulating Nucleic Acid Kit (Qiagen, Hilden, Germany, catalog number 55114) according to the manufacturer’s instructions with minor modifications to optimize recovery of low-abundance ctDNA. Briefly, plasma samples will be thawed on ice, and carrier RNA will be added to improve DNA binding to the silica membrane and increase extraction efficiency. After lysis and binding steps, cfDNA bound to the QIAamp Mini column will be washed with proprietary wash buffers to remove contaminants, and cfDNA will be eluted in 30–50 microliters (μL) of elution buffer. The concentration and quality of extracted cfDNA will be assessed using the Qubit dsDNA High Sensitivity Assay Kit (Thermo Fisher Scientific, Waltham, Massachusetts, USA) on a Qubit 4 Fluorometer. Extracted cfDNA will be stored at –80 °C and subjected to no more than two freeze-thaw cycles before analysis to maintain DNA integrity.

#### Plasma HPV ctDNA detection by droplet digital PCR and exploratory NGS

ctDNA will be quantified using a validated multiplex ddPCR assay targeting the E6/E7 regions of HPV16 and HPV18, the two most common high-risk HPV types associated with cervical cancer. ddPCR provides absolute quantification without standard curves, offering high sensitivity and precision for low-abundance targets. All analyses will be performed at the Molecular Diagnostics Laboratory, Shanghai MedTech Bio-Molecular Testing Center, using the AccuONE-R300PB chip-based system with multiplex detection of HPV16, HPV18, and a human internal reference gene (ER) to ensure sample quality and normalize DNA input. Stringent quality control measures, including no-template and positive controls, will be applied, and samples failing QC or with insufficient droplet counts will be repeated. HPV ctDNA concentrations will be calculated as copies per milliliter of plasma. ctDNA positivity will be determined according to the prespecified analytical criteria of the validated ddPCR assay, including the limit of blank (LoB), limit of detection (LOD), limit of quantification (LOQ), droplet quality metrics, and negative and positive controls. Samples with HPV16/18 E6/E7 signals reaching or exceeding the assay-defined LOD/LOQ will be classified as ctDNA-positive/detectable, whereas samples with no HPV16/18 signal or signals not exceeding the LoB will be classified as ctDNA-negative/undetectable (18, 19). Trace-positive results will be defined as low-level HPV16/18 signals above the assay background or LoB but below the assay-defined LOD/LOQ, or as low-copy signals that do not meet the predefined criteria for reliable quantification (18). These samples will be repeated when sufficient residual cfDNA is available and will be reported separately. In the primary analysis, trace-positive samples will not be considered definitively ctDNA-positive. If trace-positive results are observed, an exploratory sensitivity analysis will be conducted by reclassifying these samples as ctDNA-positive to assess whether their classification affects the primary findings. Exploratory targeted NGS will be performed in approximately 20 patients to validate ddPCR findings and explore additional tumor-specific genomic alterations. Patients with detectable or trace-positive HPV ctDNA at 3 months after CCRT, disease recurrence or progression during follow-up, or high baseline ctDNA levels will be prioritized. Approximately one-third of the NGS subset will be selected from patients with undetectable ctDNA at 3 months, balanced by FIGO stage, histological subtype, and baseline HPV16/18 status. NGS findings will be exploratory and used for assay cross-validation and hypothesis generation rather than formal subgroup inference. Detailed procedures for cfDNA extraction, assay setup, primer/probe sequences, thermal cycling, quality control, quantification formulas, and NGS methods are provided in the Supplementary Materials.

### Clinical evaluation and imaging

Treatment response will be assessed at 3 months (± 2 weeks) after completion of CCRT using pelvic MRI as the primary imaging modality, supplemented by PET-CT in selected cases based on clinical indication (such as suspected distant metastases or equivocal MRI findings). MRI will be performed using a 1.5-Tesla or 3-Tesla scanner with appropriate pelvic coils, and will include multiparametric sequences such as T2-weighted imaging in axial, sagittal, and coronal planes, T1-weighted imaging, diffusion-weighted imaging (DWI) with apparent diffusion coefficient (ADC) mapping, and dynamic contrast-enhanced (DCE) imaging. Tumor response will be evaluated according to Response Evaluation Criteria in Solid Tumors (RECIST) version 1.1, with complete response (CR) defined as disappearance of all target lesions, partial response (PR) as at least a 30% decrease in the sum of diameters of target lesions, progressive disease (PD) as at least a 20% increase in the sum of diameters of target lesions or appearance of new lesions, and stable disease (SD) as neither sufficient shrinkage to qualify for PR nor sufficient increase to qualify for PD.

Follow-up imaging will be performed every 6 months (± 4 weeks) for the first 2 years, then annually for years 3–5, or more frequently if clinically indicated based on symptoms suggestive of disease recurrence (such as abnormal vaginal bleeding, pelvic pain, or new onset of symptoms). Additional imaging studies such as chest CT, abdominal CT, or bone scintigraphy may be performed at the discretion of the treating physician if metastatic disease is suspected. All imaging studies will be reviewed by radiologists with expertise in gynecologic oncology who are blinded to ctDNA results, and findings will be documented using standardized radiology report templates. Imaging-detected disease recurrence will be confirmed by biopsy whenever feasible and clinically appropriate to obtain histopathologic confirmation.

### Outcome measures

The primary outcome of this study is progression-free survival (PFS), defined as the time from the date of initiation of CCRT to the date of first documented disease progression (according to RECIST 1.1 criteria on imaging studies or histopathologic confirmation), disease recurrence (appearance of new lesions after achieving CR), or death from any cause, whichever occurs first. Patients who are alive and progression-free at the time of analysis will be censored at the date of their last adequate tumor assessment.

Secondary outcome include: (1) Overall survival (OS), defined as the time from the initiation of CCRT to death from any cause, with patients who are alive at the time of analysis censored at the date they were last known to be alive; (2) Objective response rate (ORR), defined as the proportion of patients achieving CR or PR at the first imaging assessment (3 months post-CCRT) according to RECIST version 1.1 criteria; (3) Dynamic changes in plasma HPV ctDNA levels expressed as continuous variables (copies per mL) and categorical variables (detectable versus undetectable) at predefined time points (T0, T1, T2, T3, and follow-up time points) during and after CCRT, including assessment of ctDNA clearance kinetics (time to first undetectable ctDNA), nadir ctDNA levels, and patterns of ctDNA dynamics (rapid clearance, slow clearance, persistent detection, or reappearance after initial clearance); (4) Predictive performance of HPV ctDNA for disease recurrence, including calculation of sensitivity (proportion of patients with recurrence who have detectable ctDNA), specificity (proportion of patients without recurrence who have undetectable ctDNA), positive predictive value (PPV; probability of recurrence given detectable ctDNA), negative predictive value (NPV; probability of no recurrence given undetectable ctDNA), and area under the receiver operating characteristic curve (AUC-ROC) for ctDNA as a predictive biomarker, with 95% confidence intervals (CIs); and (5) Concordance between HPV ctDNA status and imaging-based assessment (MRI and/or PET-CT) in detecting residual disease at 3 months post-CCRT and early relapse during follow-up, assessed using Cohen’s kappa statistic and contingency table analysis. Additionally, we will explore the lead time advantage of ctDNA detection, defined as the time interval between ctDNA-detected recurrence and imaging-detected recurrence in patients who experience disease progression (20). The detailed schedule of assessments follows the SPIRIT framework and is presented in **Table 1**, which outlines all study procedures, evaluations, and time points from screening through close-out.

### Sample size calculation

The sample size was calculated based on the primary endpoint of 2-year progression-free survival (PFS), comparing patients with detectable versus undetectable plasma HPV ctDNA at 3 months after completion of CCRT (T3). Institutional historical data indicate a 2-year PFS of approximately 75% in the ctDNA-negative group, while published studies suggest a rate of approximately 50% in the ctDNA-positive group (21). Based on prior studies of HPV-associated malignancies, we conservatively estimated that 30% of patients would be ctDNA-positive at the T3 assessment. Sample size was estimated using the log-rank test for comparing survival curves between two groups: $\text{n}={\left({\text{Z}}_{1}-\alpha {/}_{2}+{\text{Z}}_{1}-\beta \right)}^{2}/\left[{\left(\ln\text{HR}\right)}^{2}\times \text{p}\left(1-\text{P}\right)\right]$.

where $Z_{1-\alpha /2}=1.96$ for a two-sided α = 0.05, $Z_{1-\beta }=0.842$ for 80% power, and *p* = 0.30 represents the proportion of patients expected to be ctDNA-positive. The hazard ratio (HR) was derived from the assumed survival probabilities ( $S_{\text{ctDNA}+}=0.50$, $S_{\text{ctDNA}-}=0.75$), yielding an estimated HR of 2.41. Using these assumptions, a minimum of 48 evaluable patients is required to achieve 80% power to detect a difference in 2-year PFS at a two-sided significance level of 0.05. Allowing for an anticipated 15% attrition rate, the total target enrollment is 57 patients. This sample size is considered feasible for recruitment at a single high-volume tertiary cancer center over the planned 2-year enrollment period and is supported by effect sizes reported in prior studies of ctDNA as a prognostic biomarker in HPV-associated cancers (21, 22).

### Statistical analysis

All statistical analyses will be performed using SPSS v27.0 (IBM Corp., USA) and R v4.3.0 or later. Baseline characteristics will be summarized using descriptive statistics: continuous variables as mean ± SD or median (IQR) depending on normality, and categorical variables as frequencies and percentages. Group comparisons (ctDNA-positive vs ctDNA-negative) will use Student’s t-test or Mann-Whitney U test for continuous variables, and χ² or Fisher’s exact test for categorical variables.

Longitudinal ctDNA changes will be analyzed using generalized estimating equations (GEE) or linear mixed-effects models to account for repeated measures, adjusted for baseline covariates such as FIGO stage, tumor size, and nodal status. PFS and OS will be estimated by Kaplan-Meier method and compared with log-rank tests; hazard ratios (HRs) with 95% CIs will be obtained from univariate and multivariate Cox proportional hazards models. Covariates in multivariate models will be checked for collinearity (VIF >5) and proportional hazards assumption (Schoenfeld residuals).

Predictive performance of ctDNA for recurrence will be evaluated using ROC curves (AUC, sensitivity, specificity, PPV, NPV); optimal cut-offs for continuous ctDNA will be determined via Youden’s index. Concordance between ctDNA and imaging will be assessed with Cohen’s kappa. Missing data<20% will be handled by multiple imputation (MICE), with sensitivity analyses including complete-case analyses. Patients lost to follow-up will be censored at the last known date. Two-sided p-values<0.05 indicate statistical significance, with multiple testing correction applied for secondary endpoints. Exploratory subgroup analyses may be conducted by FIGO stage, histology, or baseline HPV type. These subgroup analyses will be exploratory and hypothesis-generating and will not be used for confirmatory subgroup inference.

### Data management and quality control

All study data will be recorded electronically using a secure, validated EDC system (REDCap, Tianjin Medical University) with password-protected, role-based access and comprehensive audit trails. Source documents (CRFs, lab and imaging reports, medical records) will be retained on-site in accordance with GCP and institutional policies. Study personnel will undergo standardized training on eligibility assessment, study procedures, sample handling, CRF completion, and blinding, with documentation of competency. Centralized data monitoring will occur quarterly by an independent CRA, and on-site source data verification will be conducted at least twice per year. Data queries will be resolved promptly through the EDC system, with all corrections documented. An independent Data Safety Monitoring Committee (DSMC), comprising at least three experts in gynecologic oncology, radiation oncology, and biostatistics, will review safety data every 6 months or after enrollment of every 20 patients, and provide recommendations on study continuation, modification, or termination.

## Discussion

This prospective cohort study protocol establishes a systematic framework for evaluating HPV ctDNA as a dynamic biomarker in LACC patients receiving CCRT. The integration of serial ctDNA monitoring throughout the treatment continuum—from baseline through post-treatment follow-up—enables comprehensive assessment of tumor burden dynamics and early identification of molecular residual disease before clinical or radiographic manifestations become apparent. This approach addresses a critical unmet need in cervical cancer management, as conventional imaging modalities often demonstrate delayed sensitivity for residual microscopic disease and limited ability to distinguish post-treatment inflammatory changes from viable tumor tissue.

The translational implications of HPV ctDNA monitoring are substantial. First, the tumor-specific nature of HPV DNA sequences integrated into cancer cell genomes provides exceptional analytical specificity, as these viral sequences are absent from normal human cells and high-grade intraepithelial lesions (7). Second, the ability to detect molecular residual disease before radiographic progression enables potential treatment intensification or salvage therapy during the window of minimal tumor burden, when therapeutic interventions may be most effective. Recent data demonstrate detection rates of 69% at baseline in LACC patients, with strong correlation between plasma HPV ctDNA levels and tumor burden (7), suggesting that quantitative ctDNA measurements may serve as surrogate markers for tumor volume dynamics throughout treatment.

### Strengths of the study

This protocol incorporates several methodological strengths that enhance its scientific rigor and potential clinical impact. The prospective design with standardized treatment protocols and predefined blood sampling time points ensures internal consistency and minimizes ascertainment bias inherent in retrospective analyses. Serial quantitative monitoring using ddPCR technology provides high analytical sensitivity (lower limit of detection<10 copies per reaction) and precision for low-abundance targets without requiring standard curves (8). The integration of ctDNA data with comprehensive clinical, imaging, and pathological assessments enables robust multivariable analyses to determine independent prognostic value while controlling for established risk factors. Furthermore, the exploratory NGS analysis in a subset of patients facilitates validation of ddPCR findings and assessment of tumor-specific somatic mutations that may associate with treatment resistance, generating hypothesis-generating data for future mechanistic investigations.

The anticipated findings from this study may inform risk-adapted surveillance paradigms and future therapeutic strategies. Patients with rapidly cleared HPV ctDNA and sustained undetectable status may potentially undergo de-escalated follow-up with reduced imaging frequency, improving quality of life while maintaining surveillance efficacy. Conversely, those with persistent or reappearing ctDNA warrant intensified monitoring and consideration for clinical trials evaluating consolidation therapies such as immune checkpoint inhibitors, which have demonstrated efficacy in recurrent/metastatic settings (23). The recent integration of ctDNA monitoring into large randomized trials, including biomarker substudies within CALLA (durvalumab) and KEYNOTE-A18 (pembrolizumab), exemplifies the growing recognition of molecular assessment in clinical decision-making (24, 25).

### Feasibility and limitation

As a single-center study, external generalizability may be constrained by institutional practice patterns, patient demographics, and technical expertise in ctDNA analysis. Future multicenter studies across different regions, laboratories, and detection platforms will be required to externally validate the ddPCR thresholds, ctDNA clearance patterns, and predictive performance observed in this study. While the calculated sample size of 57 patients provides 80% power for the primary endpoint, subgroup analyses stratified by FIGO stage, histologic subtype, or baseline HPV type will have limited statistical power. Therefore, these analyses will be considered exploratory and hypothesis-generating, rather than confirmatory, and will mainly inform the design of future larger studies. The 24-month follow-up duration, while sufficient to capture most early recurrences, may inadequately characterize late relapses occurring beyond 2 years, particularly in patients achieving complete response. Long-term clinical follow-up will continue after completion of the planned 24-month study period, and updated analyses of long-term PFS, OS, and late recurrence patterns will be conducted in future reports. Pre-analytical variability represents another potential limitation; despite standardized protocols for blood collection, processing, and storage, factors such as hemolysis, delayed processing beyond 2 hours, or degradation during freeze-thaw cycles could influence ctDNA recovery and quantification (26). The absence of randomization precludes causal inference regarding ctDNA-guided therapeutic interventions, limiting the ability to demonstrate whether acting on ctDNA results improves outcomes compared to standard surveillance alone.

Technical challenges in liquid biopsy implementation also warrant acknowledgment. Although ddPCR offers superior sensitivity compared to traditional methods, detection rates remain imperfect, with approximately 30-40% of LACC patients testing negative at baseline despite confirmed HPV-positive tumors (27). This heterogeneity may reflect biological factors (tumor shedding kinetics, vascular invasion patterns), technical variables (HPV genotype distribution, assay design limitations), or disease characteristics (tumor size, stage). Furthermore, standardization across different platforms and laboratories remains challenging, as demonstrated by variability in detection thresholds, quality control metrics, and quantification algorithms reported across studies (28). The field requires consensus on analytical validation criteria, reporting standards, and clinical decision thresholds before widespread implementation can be achieved.

This prospective cohort study will evaluate the clinical utility of HPV ctDNA as a dynamic biomarker for treatment response and prognosis in LACC. By using standardized protocols and comprehensive outcome assessments, it aims to address key knowledge gaps, support personalized post-CCRT surveillance, identify patients at high risk of recurrence, and inform future ctDNA-guided interventional trials, advancing precision oncology in cervical cancer.

## Data Availability

The original contributions presented in the study are included in the article/**Supplementary Material**. Further inquiries can be directed to the corresponding author.
