# Supplementary material for: Plasma HPV ctDNA as a dynamic biomarker for treatment response and prognosis in locally advanced cervical cancer: protocol for a prospective cohort study
Source: Front Oncol. 2026 Jul 22;16:1844938. doi: 10.3389/fonc.2026.1844938 (PMC13438198; doi:10.3389/fonc.2026.1844938)
Supplement: Supplementary file 1 [file DataSheet1.docx]

**Droplet Digital PCR Assay**

Plasma HPV ctDNA will be quantified using a validated multiplex droplet digital PCR (ddPCR) assay targeting the E6/E7 regions of HPV16 and HPV18, with detailed procedures for cfDNA extraction, assay conditions, quality control, and analytical performance described in the Supplementary Materials.

HPV ctDNA will be quantitatively detected using droplet digital PCR (ddPCR), which provides absolute quantification without the need for standard curves and offers superior sensitivity and precision compared to quantitative real-time PCR (qPCR), particularly for low-abundance targets. The assay will target the E6 and E7 oncogene regions of HPV type 16 and HPV type 18, which are the most common high-risk HPV types associated with cervical cancer and together account for approximately 70% of cervical cancer cases globally(1). Detection of HPV16 and HPV18 ctDNA serves as a tumor-specific biomarker with high analytical specificity, as these viral DNA sequences are integrated into the tumor cell genome and are not present in normal human cells.

All ddPCR analyses will be performed at the Molecular Diagnostics Laboratory, Shanghai MedTech Bio-Molecular Testing Center, using the AccuONE-R300PB Digital PCR System (Shanghai MedTech Bio-Medical Technology Co., Ltd., Shanghai, China). This chip-based ddPCR platform partitions each reaction into approximately 20,000 independent nanoliter-scale reaction chambers, enabling precise quantification of target DNA molecules. Reagents include the 10× DigitalAmp® PCR Master Mix (Shanghai MedTech, catalog number DM-001) and Digital PCR Chips (Shanghai MedTech, catalog number DC-R300), along with sealing oil (Shanghai MedTech, catalog number SO-001) to prevent cross-contamination between chambers.

Specific TaqMan primer-probe sets will be used for multiplex detection of HPV16 E6/E7 (detected in the FAM fluorescence channel with excitation/emission at 495/520 nanometers [nm]), HPV18 E6/E7 (detected in the HEX channel at 535/556 nm), and the human estrogen receptor gene (ER) as an internal reference control (detected in the CY5.5 channel at 675/694 nm). The ER gene, a single-copy human genomic gene, serves to assess sample quality, confirm adequate DNA extraction efficiency, and normalize for input DNA quantity. Primer and probe sequences have been validated in previous studies to ensure high analytical sensitivity (lower limit of detection <10 copies per reaction) and specificity (no cross-reactivity with other HPV types or human genomic DNA).

Each ddPCR reaction will have a total volume of 15 μL comprising 1.5 μL of 10× DigitalAmp® PCR Master Mix, 0.9 μL of 20× primer-probe mix (containing forward and reverse primers at 900 nanomolar [nM] final concentration and probe at 250 nM final concentration for each target), 10 μL of extracted plasma cfDNA template, and nuclease-free water to final volume. Reactions will be loaded onto Digital PCR Chips, sealed with sealing oil, and subjected to thermal cycling. To maximize detection sensitivity and account for the low abundance of ctDNA in some samples, 10 μL of template DNA (representing a substantial fraction of the extracted cfDNA) will be used per reaction, and duplicate or triplicate reactions may be performed for each sample when DNA quantity permits.

The PCR cycling program will consist of the following steps: (1) initial denaturation at 95°C for 5 minutes (1 cycle) to activate the hot-start DNA polymerase and ensure complete denaturation of template DNA; (2) 40 cycles of denaturation at 95°C for 15 seconds followed by annealing and extension at 54°C for 25 seconds, optimized to achieve specific amplification of target sequences while minimizing non-specific amplification; and (3) a final hold step at 12°C to maintain reaction products until chip reading. Following thermal cycling, chips will be read on the AccuONE-R300PB reader, which uses a three-color optical detection system to simultaneously measure fluorescence intensity in each chamber for the FAM, HEX, and CY5.5 channels.

**Quality Control and Quantification**

Stringent quality control measures will be implemented to ensure the reliability and reproducibility of ddPCR results. Each batch of ddPCR assays (maximum 96 samples) will include at least two no-template controls (NTCs) containing all reaction components except template DNA to monitor for potential reagent contamination or cross-contamination during sample processing. NTCs must show zero or minimal positive droplets (defined as <3 positive droplets per reaction, consistent with random background noise). Any batch with NTC contamination exceeding this threshold will be repeated. Additionally, positive control samples containing known concentrations of synthetic HPV16 and HPV18 DNA fragments will be included in each batch to verify assay performance and enable inter-batch calibration if necessary.

Sample quality will be assessed by successful detection of the human internal reference gene (ER) in the CY5.5 channel. Adequate DNA extraction and sample integrity will be confirmed by the presence of ≥500 copies per mL of ER signal. Samples failing to meet this criterion will be flagged for potential preanalytical issues such as insufficient plasma volume, hemolysis, or DNA degradation, and may be excluded from analysis or re-extracted if remaining plasma volume permits.

For each sample, the total number of analyzed droplets (or chambers) must exceed 10,000 to ensure statistical robustness of quantification. Samples with fewer than 10,000 analyzable droplets due to technical issues will be repeated. The ddPCR analysis software will automatically classify each droplet as positive (containing target DNA) or negative (no target DNA) based on fluorescence amplitude thresholds established using positive and negative controls. Manual review of fluorescence amplitude plots will be performed for all samples to ensure appropriate threshold setting and to identify any anomalous signal patterns.

ctDNA concentration will be calculated in copies per milliliter of plasma using the formula:

ctDNA (copies/mL) = [measured copies per reaction × (total reaction volume / template volume)] × (elution volume / plasma input volume)

Specifically: ctDNA (copies/mL) = [measured copies × (15 μL / 10 μL)] × (elution volume / plasma volume used for extraction) × 1000

where 15 μL is the total reaction volume, 10 μL is the volume of template DNA added per reaction, elution volume is typically 30–50 μL, and plasma volume used for extraction is typically 1–4 mL. The factor of 1000 converts the units to copies per milliliter. For samples with HPV16 or HPV18 detected, the final reported value will be the sum of HPV16 and HPV18 copies per mL. A sample will be defined as positive for HPV ctDNA if the quantitative copy number of either HPV16 or HPV18 exceeds the analytical limit of detection (aLOD) of the ddPCR assay, which is 5 copies per mL of plasma (corresponding to approximately 1 copy per 2 mL of plasma based on assay validation studies)(2). Samples with detectable signal below the aLOD will be classified as "trace positive" and analyzed separately in sensitivity analyses.

**Exploratory Next-Generation Sequencing Analysis**

In a subset of patients (approximately 20–30 patients with available residual plasma samples at multiple time points), exploratory targeted next-generation sequencing (NGS) will be performed to validate ddPCR findings, assess tumor-specific somatic mutations beyond HPV status, and explore genomic alterations that may be associated with treatment resistance or disease progression. A custom-designed targeted sequencing panel covering approximately 50–100 genes frequently altered in cervical cancer (including TP53, PIK3CA, PTEN, KRAS, STK11, and others) will be used, employing hybrid capture-based enrichment followed by paired-end sequencing on an Illumina platform (Illumina, San Diego, California, USA). This exploratory analysis will generate hypothesis-generating data for future mechanistic studies but will not be used for primary outcome assessment in this protocol.

1.	Sivars L., Jylhä C., Crona Guterstam Y., Zupancic M., Lindqvist B., Nordenskjöld M., et al. Cell-free human papillomavirus DNA is a sensitive biomarker for prognosis and for early detection of relapse in locally advanced cervical cancer. Clinical Cancer Research (2024) 30:2764-71.

2.	Gupta A., Dagar G., Das S. K., Chauhan R., Shankar A., Sharma D. N., et al. Prognostic value of circulating HPV cell-free DNA in cervical cancer using liquid biopsy. Scientific Reports (2025) 15:1-9.
